# Effect of COVID-19 vaccination on transmission among healthcare workers in South Korea

**DOI:** 10.1017/ash.2022.200

**Published:** 2022-05-16

**Authors:** Jiyun Kim, Jiwon Jung, Songhee Namgung, Jihye Jung, Sun Kyung Kim, Young-ju Lim, Eun Ok Kim, Sung-Han Kim

## Abstract

**Background:** SARS-CoV-2 infection of healthcare workers (HCWs) occasionally occurs via acquisition from their colleagues. Data regarding the infection rates of HCWs with close contact and non–close contacts of HCWs are limited. In addition, the protective effect of COVID-19 vaccination against transmission between HCWs is unknown. We evaluated the infection rates of HCWs with close contact and non–close contact of infected HCWs and the effect of COVID-19 vaccination on transmission among HCWs in a tertiary-care hospital in South Korea. **Methods:** This study was performed in a tertiary-care hospital in Korea. We analyzed the COVID-19 cases and contacts among HCWs from January to December 2021. We reviewed the vaccination status of confirmed and exposed HCWs, the type of vaccination, and the infection rate according to the contact. We performed subgroup analyses in individuals who had been diagnosed since July 2021 when the δ (delta) variant became the dominant strain in South Korea. Transmission was defined based on their spatiotemporal epidemiologic association. **Results:** During the study period, 173 HCWs had COVID-19, and 2,693 HCWs were exposed to them. Among them, 18 (1.52%) of 1,186 close contacts and 13 (0.86%) of 1,507 non–close contacts had a positive SARS-CoV-2 test (*P* = .11). When the index cases had been fully vaccinated, the infection rate of close contacts was 0.85% (7 of 820), whereas the infection rate of close contacts was 3.01% (11 of 366) when the index had not been fully vaccinated (*P* = .005). However, the infection rate of non–close contacts was not different according to the vaccination status of index (0.83% vs 0.89%; *P* = .90). During the period of δ (delta) variant being dominant, the infection rate of close contacts was significantly lower when the index case had been fully vaccinated index than in cases with a non–fully vaccinated index case (0.85% vs 5.88%; *P* < .001). **Conclusions:** Transmission to colleagues was significantly lower from vaccinated HCWs than from nonvaccinated HCWs, and this finding was more significant in the era of the δ (delta) variant. Our findings support the importance of vaccination in HCWs.

**Funding:** None

**Disclosures:** None

